# When fat meets the gut—focus on intestinal lipid handling in metabolic health and disease

**DOI:** 10.15252/emmm.202114742

**Published:** 2022-04-19

**Authors:** Magdalena Wit, Jonathan Trujillo‐Viera, Akim Strohmeyer, Martin Klingenspor, Mohammed Hankir, Grzegorz Sumara

**Affiliations:** ^1^ Nencki Institute of Experimental Biology Polish Academy of Sciences Warszawa Poland; ^2^ Rudolf‐Virchow‐Zentrum Center for Integrative and Translational Bioimaging University of Würzburg Würzburg Germany; ^3^ Chair for Molecular Nutritional Medicine Technical University of Munich TUM School of Life Sciences Weihenstephan Freising Germany; ^4^ EKFZ ‐ Else Kröner‐Fresenius‐Center for Nutritional Medicine Technical University of Munich Munich Germany; ^5^ ZIEL ‐ Institute for Food & Health Technical University of Munich Freising Germany; ^6^ Department of General, Visceral, Transplant, Vascular and Pediatric Surgery University Hospital Wuerzburg Wuerzburg Germany

**Keywords:** enterocyte, fat absorption, intestine, metabolic diseases, triglycerides, Digestive System, Metabolism

## Abstract

The regular overconsumption of energy‐dense foods (rich in lipids and sugars) results in elevated intestinal nutrient absorption and consequently excessive accumulation of lipids in the liver, adipose tissue, skeletal muscles, and other organs. This can eventually lead to obesity and obesity‐associated diseases such as type 2 diabetes (T2D), non‐alcoholic fatty liver disease (NAFLD), cardiovascular disease, and certain types of cancer, as well as aggravate inflammatory bowel disease (IBD). Therefore, targeting the pathways that regulate intestinal nutrient absorption holds significant therapeutic potential. In this review, we discuss the molecular and cellular mechanisms controlling intestinal lipid handling, their relevance to the development of metabolic diseases, and emerging therapeutic strategies.

## Graphical abstract

In this review, M. Wit & colleagues discuss the molecular and cellular mechanisms controlling intestinal lipid handling, their relevance to the development of metabolic diseases, as well as emerging therapeutic strategies for the treatment of obesity and obesity‐associated diseases.

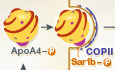

**Glossary**
The intestinal wall: The wall of the intestine consists of four layers; **mucosa** (containing epithelial cells and responsible for selective nutrient absorption), **submucosa** (supportive layer of collagen‐rich extracellular matrix), **muscular layer** (promoting gut motility), and **adventitia** (layer of loose connective tissue). The mucosa contains single‐cell layer folded in the structure termed as **Villus** which increases the absorptive surface of the intestine.
The intestinal epithelium: Multiple cell types build the intestinal epithelium and altogether originate from the stem cells in **Crypts**. Among them, **enterocytes** are the principal cell type responsible for lipid and other nutrient absorption. **Goblet** cells are responsible for the secretion of mucus to the intestinal lumen, **Paneth**, **Microfold**, and **Tuft cells** are responsible for immune response, different subtypes of Enteroendocrine cells secrete multiple hormones, including glucagon‐like peptide 1 (GLP‐1), while functions of the **Cup cells** are not well defined.

## Introduction

The global incidence of obesity continues to rise, with recent estimates that a quarter of the world’s population is affected. When considering the root causes of obesity, changes in the environment, rather than in our genetics, are largely to blame (Haslam & James, 2005). Modern jobs are generally less laborious, while a sedentary way of life has become more common (Haslam & James, 2005). Additionally, the mass production of ultra‐processed, energy‐dense foods has meant that our average daily caloric intake has increased by approximately 500 kilocalories (kcal) per day from 1970 to 2000 in the United States alone (Haslam & James, 2005). It is not simply this increase in total calories consumed that is the problem however, but rather where those calories come from that seems to matter most. Indeed, studies in mice suggest that dietary fat, as opposed to other nutrients, is the major contributor to excess calorie intake and weight gain (Hu *et al*, 2018). Moreover, clinical evidence suggests that a low‐fat diet lowers blood sugar and cholesterol more effectively than a low‐carbohydrate one (Hall *et al*, 2015). Metabolic syndrome is characterized by a chronic, low‐grade inflammation (meta‐inflammation) induced by over‐nutrition and obesity (Hotamisligil, 2006). Thus, indirectly, intestinal lipid absorption contributes to and may exacerbate this state. The key mediators of meta‐inflammation are macrophages dispersed within the adipose tissue, liver, intestine, and skeletal muscles (Li *et al*, 2018). Obesity‐associated alterations in the gut result in increased intestinal permeability and infiltration of bacteria into lamina propria (Cani *et al*, 2008; Amar *et al*, 2011), and macrophages that reside in the subepithelial layer of the intestine are activated by bacterial products (e.g. LPS), which is followed by the onset of metabolic disorders and chronic inflammation (Cani *et al*, 2008; Amar *et al*, 2011).

**Figure 1:**
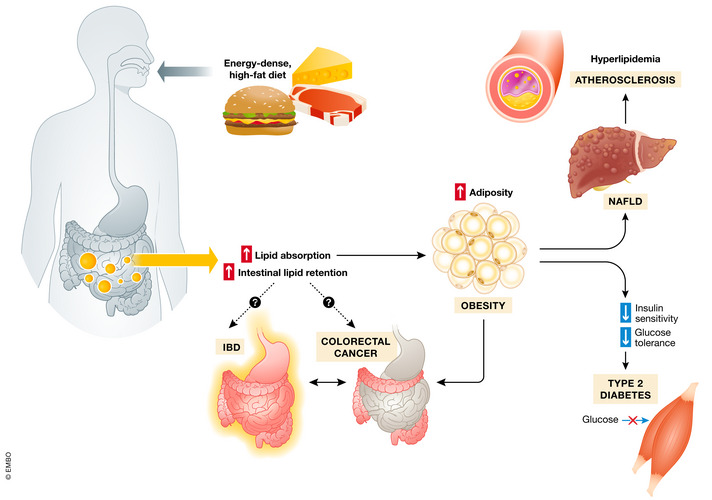
Mechanisms induced by dietary lipid overload leading to the development of metabolic diseases Food‐derived fats are efficiently absorbed in the small intestine and distributed among peripheral tissues. Supplied in excess, lipids are stored in adipose tissue thus increasing body fat mass. The imbalance between the uptake of fatty acids (FA) by the liver and insufficient lipid disposal leads to non‐alcoholic fatty liver disease (NAFLD). Systematically released from adipose tissue, FA (together with hormones, cytokines, and pro‐inflammatory factors) cause peripheral insulin resistance and contribute to pancreatic β‐cells impairment and development of type 2 diabetes. Hyperlipidemia is also an elementary risk factor for atherosclerotic plaques formation. The contribution of overload of intestinal tissue with lipids to the incidence of colorectal cancer and inflammatory bowel disease (IBD) is still not fully defined.

**Figure 2:**
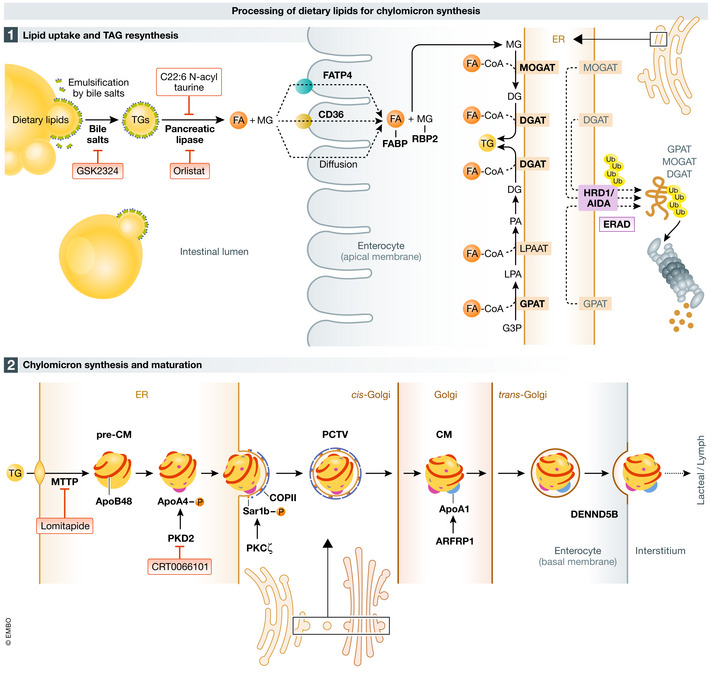
Processing of dietary lipids for chylomicrons synthesis Food‐derived lipids, in large part composed of triglycerides (TG), are emulsified by bile acid salts in the intestinal lumen to form micelles, aiding pancreatic lipase to hydrolyze TG. Final products of lipids digestion, free fatty acids (FA), and monoglycerides (MG) cross the apical membrane of the enterocyte via passive diffusion or this process is mediated by fatty acid transport protein 4 (FATP4) or CD36. Upon entering, FAs and MG are bound by fatty acid‐binding proteins (L‐FABP and I‐FABP) and retinol‐binding protein 2 (RBP2), respectively, and destined for TG re‐synthesis. FA re‐esterification is performed by subsequent action of monoacylglycerolacyl transferases (MGAT1, 2) and diacylglycerol acyltransferases (DGAT1, 2). Diacylglycerol utilized in the process can be derived from the glycerol‐3 phosphate pathway. This process can be blocked upon AIDA‐mediated endoplasmic reticulum‐associated degradation (ERAD) of MGAT2, DGAT2, and glycerol‐3‐phosphate acyltransferase 3 (GPAT3). In the endoplasmic reticulum (ER), TG are loaded into apolipoprotein B48 (APOB48)‐containing lipoprotein particle by microsomal transfer protein (MTTP); thus, a pre‐chylomicron (pre‐CM) forms, and another apolipoprotein, APOA4, is attached. Pre‐CM are transported from ER via pre‐chylomicron transport vesicles (PCTV) and fuse with the Golgi apparatus in the coat protein complex II (COPII)‐dependent manner. Mature chylomicrons (CM) are secreted via exocytosis and taken up by the local lymphatic vessel. Pharmacological agents, targeting indicated pathways, are in pink frames.

Finding ways to interfere with fat digestion would therefore appear to be a suitable approach to treat obesity and its life‐threatening comorbidities including type 2 diabetes, non‐alcoholic fatty liver disease, cardiovascular diseases, and possibly inflammatory disorders (IBD, meta‐inflammation) as well as cancer (Fig 1). As outlined briefly in Fig 2, the digestion and absorption of fat is a complex process involving a number of steps and multiple rate‐limiting enzymes (Hussain, 2014; Xiao *et al*, 2018; Ko *et al*, 2020). Also, the unique composition of the intestinal wall and whole digestive system defines the efficiency of lipid absorption (Aliluev *et al*, 2021).

From the onset, it should be mentioned that the idea of interfering with lipid digestion for therapeutic purposes (such as achieving weight loss) is not a new one. Orlistat, which limits 30% of intestinal fat absorption by inhibiting the lipases that breakdown triglycerides (TG) (Zhi *et al*, 1994), was among the earliest anti‐obesity drugs approved by the FDA in 1999 (Aaseth *et al*, 2021) but it only causes relatively modest weight loss in obese individuals after a year of the treatment (approximately 5% compared with placebo) (Sjöström *et al*, 1998). This is considerably lower than the 20–30% weight loss achieved during that time frame by the gold‐standard bariatric surgery (Maciejewski *et al*, 2016) and the stable glucagon‐like peptide 1 (GLP‐1) analogue semaglutide (Wilding *et al*, 2021). However, recent preclinical data suggest that bariatric surgery itself causes weight loss through a complex malabsorptive mechanism (Ding *et al*, 2021) attesting to the potential of targeting intestinal lipid digestion to treat obesity and other metabolic diseases. It should also be mentioned that dietary lipid overload is an independent risk factor for the development of inflammatory bowel disease (IBD) (Gruber *et al*, 2013; Luck *et al*, 2015) and colorectal cancer (Bardou *et al*, 2013). Additionally, diets rich in fat induce dysbiosis which has been implicated in the pathogenesis of gastrointestinal cancers (Font‐Burgada *et al*, 2016; Murphy *et al*, 2018). These findings have led to the idea that targeting intestinal fat processing can possibly also treat IBD and gastrointestinal cancers (Fig 1).

In this review, we concentrate on the direct molecular regulators of TG processing machinery in the small intestine and their impact on the development of multiple metabolic diseases. We start with presenting human data on how intestinal fat absorption shows major inter‐individual variability and how this could potentially be exploited therapeutically. We then extensively discuss the processes regulating the partition of the absorbed lipids between chylomicron (CM)‐mediated secretion and storage in the lipid droplet (LD). We also focus on the mechanisms regulating CM lipidation and transfer to the lymphatic system. We have largely omitted discussion on the processes regulating digestion, uptake, and re‐synthesis of TG (focusing only on the most recent concepts) and their role in the development of rare genetic diseases associated with mutations in key lipid processing enzymes, which were recently reviewed (Ko *et al*, 2020). We have also omitted other aspects of gut metabolism like the role of gut‐derived hormones, or cholesterol processing on the regulation of metabolic homeostasis. These aspects are extensively covered in (Hussain, 2014; Xiao *et al*, 2018; Ko *et al*, 2020).

## Susceptibility to weight gain: argument for the role of intestinal lipid absorption

**Figure 3:**
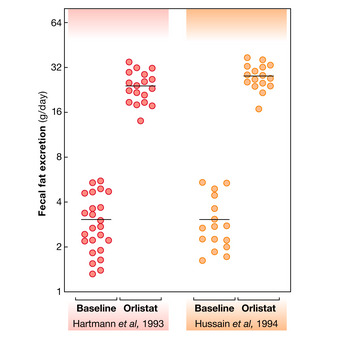
Interindividual variation in fecal fat excretion at base line and upon application of orlistat Each dot represents the amount of daily fat excretion from patients in two independent studies (Hartmann *et al*, 1993; Hussain *et al*, 1994), receiving placebo or orlistat (120 mg/day) and fed with standardized hospital diet.

**Figure 4:**
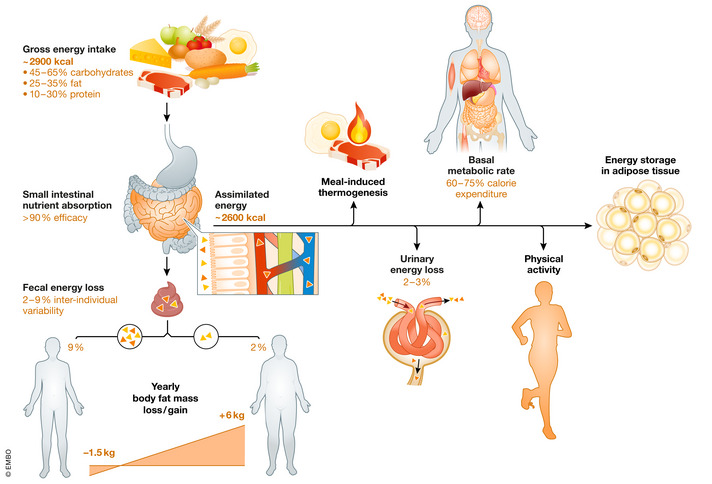
Inter‐individual variability in energy intake, excretion, and expenditure in average healthy men The figure represents a simplified overview of the daily energy balance and inter‐individual variations in the energy utilized for different physiological processes. Please note that relatively small, natural variation in energy exertion or expenditure might accumulate over a longer period (the estimation on the illustration was calculated for one year), resulting in leanness or obesity.

When it comes to human gastrointestinal physiology, surprisingly limited information is available on the absorption efficacy of ingested food, especially dietary lipids. In general, the human gut exhibits remarkably high absorption efficiency with more than 90% of gross food energy absorbed by gastrointestinal epithelial cells and transported into the body (Southgate & Durnin, 1970; Heymsfield *et al*, 1981). Correspondingly, less than 10% of gross food energy is lost by fecal excretion in healthy adults. To balance daily energy expenditure, this means that a normal weight individual metabolizing 10,800 kJ (~ 2,600 kcal) per day should ingest 12,000 kJ (~ 2,900 kcal) of gross food energy. However, substantial inter‐individual variation in absorption efficiencies exists and can range from 2 to 9% of gross food energy excreted in stool (Heymsfield & Pietrobelli, 2011; Figs 3 and 4). In one study, it was found that young men and women excreted 2.4–8.9 and 1.9–7.6 g/day, respectively, which largely exceeded their corresponding range in dietary fat intake, when fed a balanced (hospital) diet (Southgate & Durnin, 1970). A similar range was also found during baseline run‐in periods in clinical trials addressing the effects of orlistat on lipid absorption and body weight (Fig 3; Hartmann *et al*, 1993; Hussain *et al*, 1994). Although the cohorts in these studies were relatively small and a systematic assessment of inter‐individual variation is lacking, between‐subject variability in absorption efficiency appeared to be impressively consistent. Interestingly, there is preliminary evidence suggesting low fecal energy excretion in obese vs. lean subjects (Webb & Annis, 1983) raising the possibility that higher intestinal lipid absorption is a causal factor of weight gain although these findings could not be confirmed in a more recent study (Jumpertz *et al*, 2011). Nevertheless, dietary interventions or manipulation of gut microbiota composition are associated with altered intestinal absorption efficiency without diluting the individual trait (Casper *et al*, 1990; Jumpertz *et al*, 2011).

Personalized approaches can by extension be envisaged in which intestinal lipid absorption is decreased in individuals with high vs. low fecal fat (energy) excretion and who would be in a more a positive energy balance. At 12,000 kJ gross energy intake, assuming 2–3% urinary energy excretion, individuals with high vs. low absorption efficacy would gain 660 kJ (200 kcal) vs. −180 kJ (43 kcal) metabolizable energy per day. These differences in energy balance, though small on a daily basis, could potentially cumulate over the year amounting to either an increase (+6 kg) or a decrease (−1.5 kg) in total body fat mass (50.2 MJ/kg and 39.4 MJ/kg) for fat mass gain and loss, respectively (Forbes, 1990; Elia & Livesey, 1992). The substantial range of intestinal fat absorption efficiencies strongly suggests that this physiological trait is susceptible to interventions (Fig 4). However, this would require the development of a standardized methodology that would allow an assessment of intestinal lipid absorption efficiency in patients.

## Impact of gut microbiota composition and metabolism on lipid absorption

Investigating host and microbiota interactions in the regulation of lipid absorption is another emerging concept that deserves consideration. It was first reported in 2007 that germ‐free mice are resistant to diet‐induced obesity when fed a Western‐type HFD (Bäckhed *et al*, 2007). Follow‐up studies demonstrated that this phenotype is dependent on the quality and composition of the experimental HFD (Fleissner *et al*, 2010). To this end, the dietary fat source was identified as a driver for resistance with potential implications of dietary cholesterol (Kübeck *et al*, 2016). The latter study demonstrated that germ‐free mice presented attenuated diet‐induced obesity and increased fecal fat excretion when fed a HFD based on animal fat (lard), in contrast to plant fat (palm oil), while in conventional mice no differential impact of the fat source was observed. The complex interplay of diet and microbiota in regulating the efficacy of lipid absorption, metabolism, and energy balance of the host is gaining further attention. Microbiota signatures and metabolic pathways can affect these processes by different means. The contribution of the gut microbiota to host lipid metabolism and systemic host lipidome is directly measurable in different compartments like plasma and liver (Kindt *et al*, 2018), or different intestinal segments (Liebisch *et al*, 2021). HFD feeding in mice causes specific shifts in the jejunal microbiota, creating a specific HFD microbial signature. When transferred into germ‐free mice, this HFD signature increases lipid uptake not only on a HFD but also on a regular low‐fat diet, thus demonstrating a diet‐independent capability of the small intestinal microbiota to impact lipid absorption (Martinez‐Guryn *et al*, 2018). This is likely due to distinct metabolic pathways associated with altered microbiota signatures, even maintained without continued HFD feeding. Bacterial metabolites, like L‐lactate or acetate, are able to directly affect the lipid metabolism of enterocytes by inhibiting CM secretion through different mechanisms (Araújo *et al*, 2020). Moreover, short‐chain fatty acids generated by bacterial fermentation of dietary fiber enter the portal circulation and serve as precursors for hepatic synthesis of long‐chain fatty acids (Kindt *et al*, 2018). Some bacterial taxa, such as *Lactobacillaceae,* metabolize dietary polyunsaturated fatty acids (PUFA) in defense of antimicrobial toxicity. In mice fed a HFD supplemented with the omega‐6 PUFA linoleic acid, diet‐induced obesity, adipose tissue inflammation, and glucose tolerance were improved. These beneficial metabolic effects were conveyed by the hydroxylation of linoleic acid to 10‐hydroxy‐cis‐12‐octadecenoic acid (HYA). This metabolite stimulated glucagon‐like peptide 1 (GLP1) secretion from enteroendocrine cells and improved intestinal peristalsis via prostaglandin EP_3_ receptor (EP_3_), associated with lowered intestinal lipid absorption. Mono‐association of germ‐free mice with a HYA‐producing bacterial strains confirmed the beneficial microbial impact (Miyamoto *et al*, 2019).

Information regarding the human situation remains scarce, mainly due to the poor accessibility of the small intestine. Nevertheless, mechanistic murine studies pave way for new therapeutic approaches using pre‐ or probiotics. Support comes from a recent clinical trial on patients treated with *Akkermansia muciniphila*, a bacterium which improves gut barrier function and is known to be involved in lipid metabolism (Plovier *et al*, 2017; Xu *et al*, 2020), showing that it safely attenuated aspects of the metabolic syndrome (Depommier *et al*, 2019).

## Transport of lipid through the apical membrane as a target for pharmacological intervention

Despite the relatively poor profile of orlistat (due to low weight loss efficacy with accompanying side effects) mentioned earlier, the approach of reducing intestinal fat absorption to treat metabolic disease has undergone somewhat of a renaissance in recent years and several novel candidate molecules have shown promise in preclinical studies. For example, the C22 omega‐3 fatty acid derivative C22:6N‐acyl taurine (NAT) improves fatty liver in mice by reducing intestinal TG breakdown and absorption (Fig 2) (Grevengoed *et al*, 2021). The farnesoid X receptor agonist GSK2324 has also been shown to improve fatty liver in mice by reducing intestinal fat absorption through modulating intestinal bile acid composition (Fig 2) (Clifford *et al*, 2021).

When designing drugs that target intestinal fat absorption, a thorough understanding of the molecular processes involved is essential. The initial digestion of ingested TG into fatty acids (FA), monoglycerides (MG) and glycerol starts in the mouth and continues in the stomach by the action of lingual lipase and gastric lipase, respectively (Figs 1 and 2). The remaining and majority of TG digestion then takes place in the small intestine largely by the action of pancreatic lipase (Hussain, 2014). The MG and FA generated by lipases gain entrance into enterocytes by a combination of active transport and passive diffusive mechanisms. The two main proteins implicated in intestinal fatty acid uptake are fatty acid transport protein 4 (FATP4) and a cluster of differentiation 36 (CD36) (Fig 2). However, genetic experiments on mice suggest that both proteins play a minor or redundant role in intestinal lipid absorption (Goudriaan *et al*, 2002; Drover *et al*, 2005; Shim *et al*, 2009).

Studies on the regulation of membrane fluidity (referring to the viscosity which determines diffusion rate of biomolecules within the plasma membrane) have provided insight into how passive diffusion might play the dominant role in intestinal fat absorption. Lysophosphatidylcholine acyltransferase (LPCAT3) catalyzes the addition of polyunsaturated FA to lysophosphatidylcholine (LPC) to form PUFA‐containing phosphatidylcholine (PC) (which changes the membrane viscosity) (Zhao *et al*, 2008; Rong *et al*, 2013). It was found that LPCAT3‐deficient mice weaned onto a high‐fat diet die within a few weeks largely due to the reduced uptake of lipids into enterocytes (Li *et al*, 2015). Remarkably, this lethal phenotype can specifically be rescued by the oral gavage of olive oil supplemented with PCs (Li *et al*, 2015). In contrast, enterocyte‐specific deletion of LPCAT3 in mice are viable when weaned onto a low‐fat diet (Wang *et al*, 2016). These mice nevertheless show reduced serum TG and cholesterol levels, again pointing to an intestinal fat absorption defect (Wang *et al*, 2016). Indeed, when placed on a medium‐fat diet, enterocyte‐specific LPCAT3‐deficient mice exhibit weight loss associated with higher fecal TG content, reduced uptake of FA into enterocytes, and lower levels of various PC species in enterocyte membranes causing them to be less dynamic (Wang *et al*, 2016). Similar to the case for global LPCAT3‐deficient mice (Li *et al*, 2015), these defects can be rescued upon administration of polyunsaturated PC (Wang *et al*, 2016). These findings suggest that the graded inhibition of intestinal LPCAT3 could be exploited to treat obesity by reducing intestinal fat absorption through regulating enterocyte membrane fluidity.

## Intestinal TG re‐synthesis as a new strategy to fix metabolism

When FAs gain entrance into the enterocyte, they are rapidly bound to intestinal and liver fatty acid‐binding proteins (I‐FABP and L‐FABP, respectively), which shuttle them to the ER. Loss of function studies in mice have provided insight into the negative roles played by I‐FABP and L‐FABP in regulating intestinal function and metabolic health. Specifically, deletion of I‐FABP2 in APOE‐deficient mice (a model for hyperlipidemia and hypercholesterolemia) resulted in the reduction of inflammation and progression of atherosclerosis due to the improvement in intestinal barrier integrity (Zhang *et al*, 2020). Similarly, deletion of L‐FABP in a mouse model of colorectal adenomas formation resulted in a reduction of polyps size and alteration of the intestinal lipidome (Dharmarajan *et al*, 2013). Beyond their role in enterocytes, FABPs are also secreted into the general circulation. It has been shown that plasma levels of I‐FABP in humans correlate positively not only with the levels of circulating TG and cholesterol as well as the degree of atherosclerosis in the carotid artery but is also an early marker of ulcerative colitis (Wiercinska‐Drapalo *et al*, 2008; Zhang *et al*, 2020). Altogether, these findings position FABPs as potential targets for pharmacological intervention for multiple conditions.

Unlike FA, MG can have one of two fates when inside the enterocyte. They can either be degraded by the action of monoglyceride lipase (MGL) into FAs and glycerol, or can be sequestered by retinol‐binding protein 2 (RBP2) (Fig 2; Lee *et al*, 2020). Remarkably, both processes have been shown to have a major impact on whole body metabolic status. Enterocyte‐specific overexpression of MGL in mice results in decreased MG in the small intestine and weight gain (Chon *et al*, 2012), while MGL‐deficient mice gain less weight on a HFD despite increased food intake and have improved oral glucose tolerance (Douglass *et al*, 2015). On the other hand, whereas RBP2‐deficient mice have increased MG in the small intestine (similar to MGL‐deficient mice) (Douglass *et al*, 2015), they are susceptible to obesity due to reduced energy expenditure and increased food intake (Lee *et al*, 2020). This unexpected metabolic phenotype might be due to the increased release of gastric inhibitory polypeptide from enteroendocrine cells which is known to promote weight gain (Lee *et al*, 2020). These findings highlight an interesting difference with enterocyte‐specific deletion of LPCAT3 in mice on HFD, who have severely reduced food intake and body weight due to increased GLP‐1 release from enteroendocrine cells (Wang *et al*, 2016). Such studies indicate that intestinal fat digestion and absorption is tightly interconnected with the endocrine function of the digestive system which is especially relevant in the context of the development of therapies to treat metabolic diseases.

Once FA and MG reach the ER in enterocytes, they are re‐esterified by the concomitant action of monoacylglycerol acyltransferase 2 (MGAT2), glycerol‐3‐phosphate (G3P), and acyl‐CoA: diacylglycerol acyltransferases 1/2 (DGAT1/2) in enterocytes (Fig 2). Multiple studies have proven (reviewed in Ko *et al* (2020)) that while these enzymes are relevant for the development of obesity, targeting any single component of this molecular machinery is not sufficient to effectively treat metabolic diseases. Therefore, a multipronged approach might be ideal to achieve amelioration of obesity and associated diseases. Misfolded proteins in the ER are recognized by ER‐associated protein degradation machinery such as Axin interaction partner and dorsalization antagonist (AIDA) and are delivered to membrane‐associated ubiquitin ligases for their degradation. Interestingly, global and enterocyte‐specific AIDA‐deficient mice are susceptible to high‐fat diet‐induced obesity due to increased lipid absorption (Luo *et al*, 2018). These mice have markedly increased GPAT3, MGAT2, and DGAT2 (but not DGAT1) protein expression in their proximal small intestine (Luo *et al*, 2018). This is due to the loss of association between AIDA and the ubiquitin ligase HRD1, which normally ubiquitinates GPAT3, MGAT2, and DGAT2 (but not DGAT1) proteins for their subsequent degradation (Fig 2). These findings suggest that taking the opposite approach of promoting activation of intestinal AIDA could efficiently protect from the development of obesity by reducing intestinal fat absorption.

## CM formation—learning from failed past attempts

**Figure 5:**
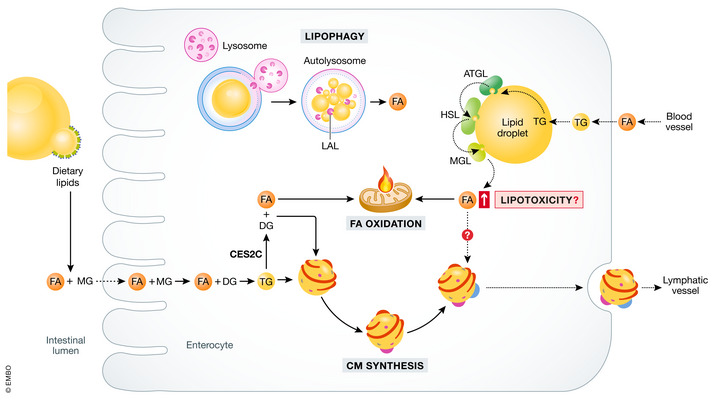
Fates of intestinal lipids mobilized from temporal storage A pool of triglycerides (TG) resynthesized from the apically delivered free fatty acids (FA) is directed for storage in cytosolic lipid droplets (LD). Hydrolysis of TG in LD is mediated by several lipases: adipose triglyceride lipase (ATGL), hormone‐sensitive lipase (HSL), and monoacylglycerol lipase (MGL). ATGL preferentially hydrolyzes lipids derived from the blood (via basolateral membrane of the enterocyte) and cleaved FAs are directed toward mitochondria for oxidation. Carboxylesterase 2c (CES2C)‐mediated TG lipolysis also provides FAs for mitochondrial energy production, while the other product, diacylglycerol (DG), is designated for chylomicron (CM) synthesis. Another pathway leading to LD catabolism is lipophagy. Lysosomal acid lipase (LAL) digests TG within autophagosomes and released FA serve as a substrate in various metabolic pathways. Hypothesized toxicity of FA extensive LD mobilization results in elevated FA concentration—potential toxic outcomes of that event are discussed in the relevant chapter.

Upon re‐synthesis in the ER, TGs can be designated for secretion by enterocytes in lipid‐loaded CM, or temporarily stored in the form of LD (Figs 2 and 5). CM are highly complex particles composed of lipoproteins, TGs, cholesterol, phospholipids, and other lipid species. The biosynthesis of CM starts with the formation of a nascent CM (pre‐CM) which is facilitated by the action of microsomal triglyceride transfer protein (MTTP) (Hesse *et al*, 2013; Mansbach & Siddiqi, 2016). MTTP regulates the translocation of apolipoprotein B48 (APOB48) into the ER and therefore the beginning of lipoprotein formation (Fig 2). Furthermore, MTTP modulates the transfer of lipids into the newly formed pre‐CM (Hussain *et al*, 2011; Hussain, 2014). Homozygous mutations in MTTP and APOB48 cause severe forms of hypolipidemia associated with multiple developmental defects. Notably, Villin‐Cre driven conditional MTTP deletion in intestinal epithelial cells exacerbates chemically induced colitis and increased associated tumor burden (Xie *et al*, 2013). The MTTP inhibitor (lomitapide) is also approved for the treatment of inherited hyperlipidemias like homozygous familial hypercholesterolemia and familial chylomicronemia syndrome, but its usage is associated with severe side effects and therefore is not recommended for the treatment of obesity (Ko *et al*, 2020). Limiting MTTP inhibition to enterocytes however might represent a more promising strategy for the treatment of obesity and hyperlipidemia. Indeed, deletion of PRAP1, which mediates the interaction between TG and MTTP to stimulate the loading of TGs into pre‐CM and is largely expressed in the intestine, decreases the formation and lipidation of APOB lipoproteins resulting in lower TG absorption (Peng *et al*, 2021).

Apolipoprotein A4 (APOA4) is another important apolipoprotein found in CM. Although it is mostly produced by the enterocytes from where it is released with chylomicrons, it can also be found in other lipoproteins (HDL, VLDL, and CM remnants) and in its free form in plasma. This versatility of APOA4 suggests that it might be a key player in the interaction between different lipoproteins as well as in their metabolism in peripheral tissues (Wang *et al*, 2015). Elevation of APOA4 is associated with increased lipid absorption from the diet, TG packaging, and CM size (Gonzalez‐Vallina *et al*, 1996). However, another study found that knockout mice for APOA4 presented with decreased TG and cholesterol in plasma but increased CM size (Kohan *et al*, 2012). These results suggest that the APOA4 protein abundance does not simply correlate with net lipid absorption. In line with this idea, a recent study showed that the lack of protein kinase D2 (PKD2) in mice results in increased circulating levels of APOA4 but decreased lipid absorption and body weight gain in association with a healthier metabolic profile in these mice and enrichment of the microbiota with anti‐obesogenic members of *Bacteroides* spp. in the intestine (Trujillo‐Viera *et al*, 2021). Since no difference was found in the amount of APOB48 (reflecting the abundance of CM), and PKD2 directly phosphorylates APOA4, the lack of this post‐translational modification might be responsible for the observed phenotype (Trujillo‐Viera *et al*, 2021). Hypothetically, PKD2‐dependent phosphorylation of APOA4 might promote maturation/lipidation of the CM by increasing the retention time in the ER. The use of a small‐molecule inhibitor of PKD2 as well as PKD1 and PKD3 (CRT0066101), also allowed a localized inhibition of this pathway in the small intestine, thereby decreasing intestinal lipid absorption to ameliorate obesity and restore insulin sensitivity. Notably, activity of PKD2 in the small intestine of obese patients was found to correlate with plasma TG levels (Trujillo‐Viera *et al*, 2021). These results demonstrate that regulation of abundance and enzymatic modifications of APOA4 might be of therapeutic use for the treatment of obesity and hyperlipidemia. Moreover, in addition to the functional role of APOA4 in lipoprotein interaction and metabolism, central APOA4 is considered to act as a satiety signal by increasing the activity of hypothalamic proopiomelanocortin neurons (Yan *et al*, 2016). Thus, the multiple actions of APOA4 need to be fully investigated to exploit its full potential as a target for the treatment of metabolic diseases.

Upon assembly of the pre‐CM, pre‐CM transport vesicles (PCTV) are formed which transport lipids from the ER to the *cis‐*Golgi in a coat protein II‐dependent manner (Fig 2; Mansbach & Siddiqi, 2010). Once inside this organelle, pre‐CM undergo lipidation and addition of another lipoprotein, APOA1. Trafficking of CM through the Golgi requires also an action of golgins and GTPases, which regulate the fate of this cargo (Lu *et al*, 2004; Zahn *et al*, 2006). In the case of CM, the GTPase ADP‐ribosylation factor‐related protein 1 (ARFRP1) seems to be of high importance for lipidation, addition of apolipoprotein A1 (APOA1), and the release of mature CM (Fig 2).

The mechanism involves recruitment to the Golgi of ADP‐ribosylation factor‐like 1 (ARL1) by activated ARFRP1where it binds to Golgin‐245 and another GTPase (Rab2) to promote CM lipidation and subsequent transport into the Golgi (Jaschke *et al*, 2012; Hesse *et al*, 2013). Intestinal deletion of ARFRP1 results in growth retardation reduced TGs in plasma, reduced lipid absorption, and a decrease in the secretion of APOA1 (Jaschke *et al*, 2012). These studies demonstrate that alterations in Golgi processing might also influence the size of CM and the efficiency of lipid absorption from the diet.

The last steps of CM release from enterocytes are less well studied. However, the trafficking of these secretory vesicles seems to be regulated by the action of a transmembrane protein belonging to the differentially expressed in normal and neoplastic cells (DENN) family, specifically DENND5B (Fig 2). These DENN proteins have activity toward Rab GTPases and therefore are involved in intracellular vesicle transport (Marat *et al*, 2011). Mice with DENND5B deficiency present with improved metabolic homeostasis. In particular, these mice have decreased absorption of TG in the intestine, reduced body weight, and lower levels of cholesterol in the blood (Gordon *et al*, 2019). Analysis of the small intestine in DENND5B‐deficient mice using electron microscopy revealed a defect in the fusion of CM secretory vesicles with the plasma membrane at the basolateral side thereby interfering with their ability to reach the lamina propria (Gordon *et al*, 2019). In addition, gastrointestinal water absorption across epithelia and endothelia appears to be crucial for the efficacy of lipid absorption, as suggested by metabolic phenotyping in aquaporin 1 (AQP1) knockout mice (Ma *et al*, 2001). Mice lacking AQP1 are viable and develop normally on a regular low‐fat chow diet but exhibit steatorrhea, increased fecal lipase activity and do not gain body weight when fed a high‐fat diet, particularly at a young age. This malabsorption of dietary fat may be partially due to impaired CM transport into lacteals in the absence of AQP1. More studies are necessary to investigate the detailed mechanisms behind these last steps of CM release and transport into the lymph and their roles in the regulation of intestinal lipid absorption as well as the therapeutic potential of targeting the critical molecules involved in this process.

## Lipid droplets in enterocytes as a possible link between lipid homeostasis and inflammation

In the postprandial period, most of the lipids absorbed by the intestinal epithelium are designated for CM and a pool of TG are stored in the form of LD. Accumulated lipids can be mobilized at later times and serve as substrates for CM synthesis or β‐oxidation (Fig 5). Similar to other cell types, intestinal LD have a neutral lipid core surrounded by a phospholipid monolayer with an array of coating proteins which are known to orchestrate the synthesis and catabolism of LD. Importantly, the protein composition of LDs in enterocytes present unique characteristics compared to other cell types (Beilstein *et al*, 2016).

The dynamic nature of intestinal LD is reflected by their rapid growth and subsequent depletion in response to an oral lipid challenge (Zhu *et al*, 2009). Chronic overload with dietary lipids in mice triggers the re‐establishment of the protein composition of LD with accompanying greater size of these structures upon acute fat ingestion compared with lean mice (D’Aquila *et al*, 2019). Partial redirection of dietary TG for temporal storage instead of its direct incorporation into CM might potentially explain the observed phenomenon of reduced lipid absorption rate in the postprandial period in HFD fed mice and might serve to protect the intestinal epithelium from FA‐induced toxicity (Listenberger *et al*, 2003).

One of the best‐characterized LDs scaffold proteins are the perilipin (PAT domain) family members (perilipin 1–5). The only PAT proteins identified in murine intestinal mucosa are perilipin 2 (ADRP, adipophilin, Plin‐2) and perilipin 3 (TIP47, Plin‐3) and both are more abundant and colocalize with LDs upon dietary fat challenge (Lee *et al*, 2009). Plin‐2 associates with enterocyte LDs formed upon chronic high fat feeding but is not detected in the intestine of lean or challenged mice (Lee *et al*, 2009). Furthermore, Plin‐3 is considered as relevant for LD biogenesis, while Plin‐2 role is to stabilize already formed LDs (Wolins *et al*, 2005). However, how the impaired function of enterocyte perilipins affect TG partitioning between CM, FA oxidation and storage in LD is unclear. No genetic deletion of Plin‐3 has been generated so far to investigate its impact on gut metabolism, while Plin‐2 has been studied in mice using the global knock‐out. These mice are protected from HFD‐induced obesity, have reduced food intake, present with higher physical activity and beiging of white adipose tissue due to upregulation of uncoupling protein 1 expression (McManaman *et al*, 2013). However, the impact of Plin‐2 on lipid absorption is not clear (McManaman *et al*, 2013).

Mobilization of TGs from their storage in LD requires their hydrolytic decomposition by the lipases associated with LDs (Fig 5). This process is initiated by adipose triglyceride lipase (ATGL), and its co‐activator comparative gene identification‐58 (CGI‐58) that hydrolyses TG into DG and FA, hormone‐sensitive lipase (HSL) which hydrolyzes cholesterol esters and DG to MG and FA, and monoacylglycerol lipase that finalizes the process by degrading MG into glycerol and FA (Grabner *et al*, 2021). Intestine‐specific ATGL knockout (ATGL iKO) mice present massive lipid accumulation in LD of intestinal epithelium (Obrowsky *et al*, 2013). Interestingly, intestinal ATGL deficiency results in down‐regulation of PPARα target genes that promote cholesterol absorption and metabolism. However, increased TG retention in the mucosa was not followed by diminished lipid absorption upon intragastric trioleate administration suggesting that FAs derived from ATGL‐mediated hydrolysis of TG are not dedicated as substrates for the secretory pathway (Obrowsky *et al*, 2013). On the other hand, mice carrying deletion of one allele of the CGI‐58‐encoding gene specifically in the intestine have decreased postprandial plasma TG and cholesterol concentration and enlarged LD even in the fasting state indicating that CGI‐58 is a positive regulator for CM secretion independent on ATGL function (Xie *et al*, 2014). This hypothetical distinct function should be determined in future studies.

According to recent findings, it is more likely that ATGL and CGI‐58‐dependent catabolism of LD is critical for the release of basolaterally absorbed FAs from LD that are subsequently shuttled to mitochondria and utilized for energy production (Korbelius *et al*, 2019). Contrary to these data are the results from a study on the role of Golgi reassembly‐stacking protein of 55 kDa (GRASP55) protein in LD targeting of ATGL in the small intestine (Kim *et al*, 2020). GRASP55 is the Golgi‐resident protein involved in secretory pathways of multiple cargos that bypass the Golgi (Gee *et al*, 2011) and its deletion in mice leads to reduced CM secretion and abnormally large LD formation which systemically results in resistance to obesity and improved insulin sensitivity (Kim *et al*, 2020). Massive lipid accumulation in the midgut was also found in Drosophila, proving an evolutionarily conserved function of the GRASP55 (Kim *et al*, 2020). Altered dietary lipid metabolism was found to be associated with reduced expression levels and impaired trafficking of ATGL and MGL from the Golgi to LD surface which explains diminished TG supply for CM synthesis, according to the study.

ATGL and other lipases might also play a role in release of pro‐inflammatory lipid compounds from LD. This proposal comes from observations in adipocytes in which β‐adrenergic stimulation activates lipolysis and cyclooxygenase‐2 (COX‐2) expression with the latter being responsible for elevated eicosanoids production from arachidonic acid (Gartung *et al*, 2016). c‐Jun N‐terminal kinase (JNK)/nuclear factor kappa‐light‐chain‐enhancer of activated B cells (NF‐κB) signaling pathway can be also activated by HSL action, and *in vivo*, pharmacological inhibition of the lipase prevented the upregulation of COX‐2 and macrophage infiltration to adipose tissue (Gartung *et al*, 2016). Further, Plin‐1 interferes with ATGL to downregulate lipolysis and is essential for limiting eicosanoid production and macrophage infiltration to adipose tissue (Gartung *et al*, 2016). Increased COX‐2 activity in inflamed tissue is well documented in human subjects with colorectal cancer or IBD (Wang & DuBois, 2010). The hypothetic link between this condition and ATGL/HSL‐mediated lipolysis activity in the intestine has not been tested so far and given the analogical function of Plin‐1 and 2 as negative regulators of ATGL activity, the involvement of Plin‐2 in colorectal cancer or IBD onset or progression might be also speculated.

Deletion of second major lipase, HSL in the intestine (HSL iKO) is not followed by alterations in plasma TG concentrations in mice fed a chow or high‐fat/high‐cholesterol diet (Obrowsky *et al*, 2012). In spite of reduced levels of HMG‐CoA synthase (HMG‐CoS) and HMG‐CoA reductase (HMG‐CoR), two enzymes being in charge of cholesterol synthesis, in HSL iKO, it does not counteract the cholesterol overload and leads to an increase in plasma cholesterol concentration and CE accumulation in the small intestine upon high‐fat/high‐cholesterol feeding or intragastric cholesterol load (Obrowsky *et al*, 2012).

The catabolic pathway of TG derived from cellular storage is finalized by the action of MGL, the impact of MGL on lipid absorption was discussed in previous chapter. MGL is also a negative regulator of 2‐arachidonoyl glycerol (2‐AG). 2‐AG, similar to other endocannabinoids, exerts anti‐inflammatory properties (Maccarrone *et al*, 2015). In parallel to hydrolytic degradation of this agent, MGL contributes to the synthesis of pro‐inflammatory lipid compounds such as eicosanoids and lysophophospholipids, thus exacerbating inflammation and promoting tumorigenesis (Nomura *et al*, 2010, 2011). The potential link between MGL activity and the risk of IBD is a missing gap and requires to be filled in future studies.

TG hydrolysis in enterocyte might be supported by carboxylesterase 2c (CES2c) (Maresch *et al*, 2019). Proteins of carboxylesterases family are known mainly as enzymes involved in detoxification and metabolism of prodrugs (Hatfield *et al*, 2016). However, human and murine CES2/Ces2c can also act as potent TG and DG hydrolases involved in the development of obesity and fatty liver disease (Maresch *et al*, 2019). Intestine‐specific *Ces2c* overexpression promotes enhanced FA oxidation in enterocytes due to higher availability of FA from Ces2c‐mediated hydrolysis of TG and DG, while the generated DG and MG are destined for re‐esterification and incorporation into CM thus leading to enlarged particles’ size (Maresch *et al*, 2019). Although the overall fat absorption is not affected, mice carrying the Ces2c transgene are resistant to obesity and its comorbidities, as exemplified by NAFLD. Interestingly, declined *CES2* mRNA expression was identified in colon biopsies from patients affected by ulcerative colitis. The expression pattern of *Ces2c* in the colon was recapitulated in mice treated with dextran sulfate sodium (DSS) to induce colitis (Chalhoub *et al*, 2021). Taken together, presented data imply a critical role for CES2/Ces2c in regulation of TG hydrolysis in the small intestine and, most importantly, link intestinal lipid homeostasis with obesity and inflammatory bowel disease.

Another player coordinating enterocyte lipid storage is the milk fat globule‐EGF factor‐8 (Mfge8), a glycoprotein originally described as milk fat globule membrane compound. Through the interaction with its ligands, αvβ3 and αvβ5 integrins, Mfge8 promotes the hydrolysis of lipids stored in enterocyte LD and simultaneously the uptake of FA (Khalifeh‐Soltani *et al*, 2016). This bi‐modal mechanism is responsible for impaired lipid release to the extracellular space in enterocytes isolated from *Mfge8‐*deficient or *αvβ3*/*αvβ5‐*deficient mice which are due to reduced FA uptake and entrapment of those that are taken up in LD. The reduction of TG hydrolase activity in intact intestinal tissue from *Mfge8‐*deficient or *αvβ3*/*αvβ5‐*deficient mice by around 50% is strikingly greater compared to what is observed in animals carrying ATGL deletion (Obrowsky *et al*, 2013) or deletion of CGI‐58 (Xie *et al*, 2014) underscoring the relevance of Mfge8 in the breakdown of LD in enterocytes. The TG hydrolase(s) controlled by Mfge8/integrin axis has not been discovered yet but it is known that its activation is realized via a PI3 kinase/mTORC2 pathway. What is more, due to the regulatory role in inflammation, Mfge8 was found to ameliorate inflammatory cytokine profile in colon tissues of mice suffering from DSS‐ or trinitrobenzene sulfonic acid‐induced colitis, counteract their weight loss and colon shortening (Zhang *et al*, 2015). Although it is likely that these outcomes result from Mfge8/integrin ligation signaling restricted to immune cells only, the involvement of LD catabolism cannot be ruled out at this stage and is an interesting option to investigate.

Lipophagy, a form of autophagy, is an alternative pathway that leads to the breakdown of LD and occurs within lysosomes (Khaldoun *et al*, 2014). Dietary lipid influx into intestinal absorptive cells triggers a rapid, autophagic response through targeting of LD to lysosomes and inhibition of this process in mice results in accumulation of TG and cholesteryl esters (Khaldoun *et al*, 2014). TG degradation in lysosomes is mediated by lysosomal acid lipase (LAL) and mice deficient in the enzyme also accumulate more TG and cholesteryl esters in lysosomes of the small intestine, recapitulating the phenotype seen in human patients affected by Wolman disease and cholesteryl ester storage disease (Porto, 2014). Lowered LAL activity was also noted in patients with NAFLD that further decreases with worsening to non‐alcoholic steatohepatitis (NASH) (Baratta *et al*, 2015). These data convincingly establish LAL as another lipase controlling the turnover of LD and lysosomes as platforms for lipophagy performance.

Although perceived for a long period as inert organelle‐like particles, currently, LD are regarded as highly dynamic, and—apart from lipid storage—functionally relevant for various biological processes. Due to the dual role of enterocytes in dietary lipid handling, that is, their absorption and storage, the biology of enterocyte LD requires re‐exploration. According to data collected in the recent decade, LD might be a target for manipulating net lipid absorption through the intestinal wall, yet the major enterocyte lipase is not known. The link between LD metabolism and intestinal diseases such as IBD or colorectal cancer is only hypothetical and requires further investigation.

## Post‐secretion transport of CM—a new space for pharmacological intervention

Upon crossing the basolateral membrane of enterocytes via exocytosis, CM accumulate in the intercellular space. There are then two significant but often overlooked barriers that CM must cross before being taken up by lymphatics: the basement membrane and the lamina propria (Zhou *et al*, 2020). The gut basement membrane physically separates epithelial cells from the underlying lamina propria and is comprised of various macromolecules including collagens, laminins, proteoglycans, and structural as well as adhesion proteins. These macromolecules form a complex interconnected mesh with pores ranging in size from 10 to 130 nm—too small to allow passage of CM (Zhou *et al*, 2020). While not yet formally shown, it is thought that the accumulation of chylomicrons in the intercellular space after fat ingestion causes the basement membrane to bulge and temporarily rupture. This then allows CM to infiltrate the lamina propria (Zhou *et al*, 2020). The increased pressure in the intercellular space from the accumulation of CM also places a strain on the seal between intestinal epithelial cells, known as the intestinal epithelial barrier, which is comprised of multiprotein complexes called tight junctions, adherents junctions, and desmosomes (Schlegel *et al*, 2021). Consequently, ingestion of fat also leads to increased intestinal permeability (Kvietys *et al*, 1991) thereby providing a paracellular route for luminal factors including TG and FAs to the basement membrane.

The lamina propria is a complex protective structure comprised of various cell types including immune cells, neurons, glial cells, fibroblasts, and smooth muscle cells which together form a defensive barrier of approximately 50 µm in width that CM must finally cross before reaching their lymphatic destination. The usually gel‐like consistency of the lamina propria during non‐absorptive conditions becomes more fluid‐like during fat ingestion. This is achieved, in part, by the release of histamine from mast cells which increases vascular permeability and results in hydration of the lamina propria (Ji *et al*, 2012). During this process, average lamina propria pore size increases 4‐fold from 250 A to 1,000 A which is large enough for the passage of CM (Zhou *et al*, 2020). In turn, lymphatic flow rate determines the convective exit of CM from the lamina propria to lacteals (Zhou *et al*, 2020).

## Post‐secretion transport of CM—a new space for pharmacological intervention

**Figure 6:**
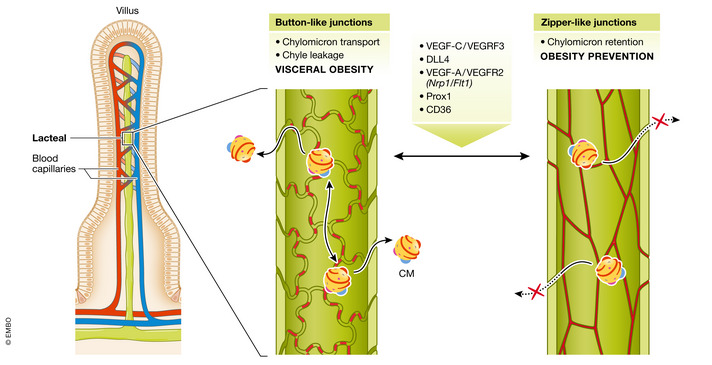
Lacteal permeability determines fat absorption Two types of cell–cell junctions between lymphatic endothelial cells (LECs) provide the lacteal’s integrity: open, “button‐like” junctions, which facilitate chylomicrons (CM) uptake from the extracellular matrix, and tight, “zipper‐like” junctions inhibiting CM entry. Transition of zipper‐to‐button junctions results in increased intestinal fat absorption and promotion of obesity, and vice versa, junctions “zippering” prevents CM uptake into the lacteal lumen and is obesity‐protective. Remodeling of junctions type in LECs is controlled by the enlisted group of factors. Mode of action of each is described in chapter **Post‐secretion transport of CM—a new space for pharmacological intervention** VEGF, vascular endothelial growth factor*; VEGFR,* vascular endothelial growth factor receptor; DLL4, Notch ligand delta‐like 4; *Nrp1*, neuropilin 1‐encoding gene; *Flt1*, VEGFR1‐encoding gene; Prox1, prospero‐related homeobox 1; CD36—cluster of differentiation 36.

Lymphatic vessels play a pivotal role in dietary fat absorption as they serve as an exclusive route of transport of CM from the intestine (Fig 6). Each intestinal villus is equipped with one or two, blind‐ended lymphatic capillaries, termed as lacteals. Lymph from lacteals is drained into mesenteric lymph nodes, subsequently transported via collective ducts to cisterna chyli and the thoracic duct directly delivers lymph to the bloodstream at the level of the subclavian vein, where the circulatory and lymphatic systems meet (Bernier‐Latmani & Petrova, 2017).

Lacteals present a unique adult vessel phenotype as their lymphatic endothelial cells (LECs) maintain a high proliferation rate reflecting constant vessel remodeling, while in general, lymphatics are quiescent structures (Bernier‐Latmani & Petrova, 2017). Vascular endothelial growth factor‐C (VEGF‐C), which is a predominant lymphangiogenic factor, acts via activation of vascular‐endothelial growth factor receptor 3 (VEGFR‐3) expressed by LECs. Inducible global *Vegfc* deletion in adult mice leads to progressive lacteal atrophy and LEC loss, without affecting the lymphatic vasculature in other organs (Nurmi *et al*, 2015). As a consequence, VEGF‐C‐depleted mice are resistant to HFD‐induced obesity, excrete more lipids with feces while their food intake is unchanged in comparison with control littermates (Nurmi *et al*, 2015). *Vegfr3* deletion results in a similar phenotype (Nurmi *et al*, 2015) and furthermore, mice carrying an inactivating mutation in VEGFR‐3 tyrosine kinase motif present lower intestinal concentration of nitric oxide (NO) which is essential for CM release from the enterocyte (Hsieh *et al*, 2015). Future studies should address the precise role of VEGFR3 in NO synthesis. A known downstream target of VEGF‐C—VEGFR3 and VEGFR2 signaling is Notch ligand delta‐like 4 (DLL4) (Bernier‐Latmani *et al*, 2015). Its genetic inactivation in LECs leads to lacteal regression and impaired dietary fat absorption (Bernier‐Latmani *et al*, 2015). Taken together, targeting VEGF‐C—VEGFR3 axis might be a strategy for preventing obesity. However, it has to be noted that lacteals are more resistant to VEGFR‐3 inhibition than blood vessels, as administration of sunitinib, an inhibitor of the receptor, affected only the blood vessel density of the villus (Nurmi *et al*, 2015). Thus, drugs of higher affinity toward intestinal LECs VEGRF3 and targeting of VEGFC can be considered in the future.

Another approach to reduce excessive lipids uptake by lacteals is by increasing the zippering of junctions in LECs which is controlled by vascular endothelial growth factor (VEGF‐A) signaling. Physiologically, VEGF‐A binds primarily to its decoy receptor vascular endothelial growth factor receptor 1 (VEGFR‐1) (*Flt1*). Another protein binding VEGF‐A is neuropilin 1 (NRP1) which functions as a VEGFR‐2 co‐receptor and regulates endothelial cell permeability. Simultaneous deletion of *Flt1* and *Nrp1* in both, blood and lymphatic endothelial cells, resulted in increased VEGF‐A—VEGFR‐2 signaling and transition of button‐like to zipper‐like junctions in lacteals and opposite rearrangement in blood endothelial cells (Zhang *et al*, 2018). Tighter junctions provided CM malabsorption and resistance to high‐fat diet‐induced obesity. As proposed, NRP1 is a novel decoy receptor for VEGF‐A and together with FLT1 expressed by villus blood endothelial cells serve to limit VEGF‐A‐VEGFR2 signaling and thus allow button‐like junctions' maturation in lacteals (Zhang *et al*, 2018). Future studies should address whether enhancement of VEGF‐A–VEGFR2 signaling in villi is applicable and sufficient to protect against obesity in humans.

On the other hand, dysfunctional lymphatics are known to contribute to the onset of obesity, type 2 diabetes, and age‐related diseases (Cifarelli & Eichmann, 2019). First evidence highlighting the importance of lacteal architecture in the etiology of obesity was demonstrated in mice deficient for one allele of *Prox1* (Harvey *et al*, 2005). Observed leakage of chyle in these mutants is the most prominent observed within the mesenteric region, where hypertrophic adipocytes primarily accumulate, and fat mass increase is proportionate to lymph‐leakage grade (Harvey *et al*, 2005). More recently, CD36 expressed by intestinal LECs was found to optimize intestinal lymphatic vessels integrity and protect against visceral obesity (Cifarelli *et al*, 2021). Lacteals of LEC‐specific CD36‐deficient mice have fragmented zipper‐like VE‐cadherin junctions and develop obesity. Disruption of VE‐cadherin junctions leads to leakage of chyle from mesenteric lymphatics to the abdominal cavity where it triggers adipogenesis (Cifarelli *et al*, 2021). This is in line with the previously proposed pro‐adipogenic role of lymph upon abnormal lymphatics leakiness (Harvey *et al*, 2005). Mechanistically, CD36 in LECs acts via VEGF‐C‐mediated activation of VEGFR‐2 and AKT signaling that independently provide junctions' tightness and stability (Zhang *et al*, 2018).

Finally, it should be noted that the process of CM transport into the lacteal lumen might require certain CM‐dependent prerequisites. A study from Van Dyck *et al* (2007) established that transcription factor pleomorphic adenoma gene‐like 2 (PlagL2) is relevant for CM modifications that enable its uptake by the lacteal (Van Dyck *et al*, 2007). Mice lacking PlagL2 die from postnatal wasting due to fat malabsorption. These mice synthesize CMs that can exit the enterocyte but fail to enter the lacteal and aggregate between the vessel and lamina propria. As PlagL2 expression is limited only to enterocytes and expression of several genes involved in metabolism and cargo transport in mutant mice was altered, these stand as candidates determining CM specific properties that are necessary for recognition by the lacteal (Van Dyck *et al*, 2007).

In summary, research performed in the last decade has revolutionized the perception of lacteals role in lipid absorption from the intestine. However, beside unraveling physiological aspects based on animal models, further studies are needed to determine the relevance of lymphatics as a target to decrease lipid absorption and therefore combat obesity and related diseases in humans.

## Concept of targeting gut metabolism against obesity is clinically proved

As recent advances in the development of pharmacological strategies to combat obesity have been extensively revised (Müller *et al*, 2022), we will focus this chapter only on approaches directly connected with intestine function.

The concept of combating obesity by targeting the intestine originated in the late ‘90s with orlistat, an inhibitor of pancreatic lipase. In clinical trials, orlistat achieves weight loss of up to ~ 10% in combination with a hypocaloric diet during the first year of the treatment, which then drops to ~ 5% on a eucaloric diet in ~ 57% of patients during the 2^nd^ year (Sjöström *et al*, 1998). As orlistat acts in the intestinal lumen and is minimally absorbed, systemic adverse effects are negligible. Gastrointestinal (GI) symptoms include fatty/oily stool, frequent defecation, fecal incontinence, and malabsorption of fat‐soluble vitamins which may require supplementation (McDuffie *et al*, 2002). The long‐term use of the drug thus needs careful monitoring with respect to efficacy and side effects.

Despite maintained interest in the molecular control of intestinal lipid handling with regard to obesity management in academic research, only a modest percentage of efforts in the field has found its way into clinical practice. One of the partially successful attempts is lomitapide, an inhibitor of both intestinal and hepatic MTTP, which impairs chylomicron and VLDL lipidation and is approved for the treatment of homozygous familial hypercholesterolemia, a rare disease caused by genetic mutations impairing lipid clearance from the blood by peripheral tissues (Perry, 2013). However, the drug is excluded as an anti‐obesity agent as it comes with major side effects including liver steatosis which may even progress to steatohepatitis and fibrosis. An alternative to lomitapide could be JTT‐130 which exclusively targets intestinal MTTP and effectively ameliorates liver damage in preclinical studies (Aggarwal *et al*, 2005; Hata *et al*, 2011), but it has not been evaluated in clinical trials so far. Benefits from the inhibition of DGAT1 with pradigastat were shown in patients with familial chylomicronemia syndrome (a rare genetic disease characterized by high blood TG levels) in a pilot study (Meyers *et al*, 2015). Over 3 weeks, it led to a 70% reduction in fasting TG, as well as a substantial decrease in postprandial APOB48 and TG. Adverse effects of pradigastat from GI tract were mild and transient. Other pharmacological inhibitors of enterocyte acyltransferases, for example, JTP‐103237 for MGAT2 (Okuma *et al*, 2015) and PF‐04620110 for DGAT1 (Dow *et al*, 2011, 2013) have again shown promise in suppressing weight gain in preclinical studies, but are still well beyond the stage of testing in humans. When considering the concept of counteracting obesity by targeting intestinal lipid digestion/absorption/processing, a major challenge is whether the use of this class of drugs is associated with adverse GI effects (steatorrhea). The experience of lomitapide indicates that the blockade of CM synthesis alone is insufficient to avoid them. The question then arises if these side effects be avoided by, for instance, re‐directing TG fate in enterocytes toward storage in LD or mitochondrial FAO? If so, how would such manipulations affect the functionality of epithelial cells?

The current gold‐standard treatment for obesity is unquestionably bariatric surgery, as evidenced by the long‐term 30–40% weight loss in clinical trials (Maciejewski *et al*, 2016) with decreased mortality from cardiovascular disease or cancer by 30 and 23%, respectively (Carlsson *et al*, 2020). Although the surgical approach was originally intended to help lose weight through restricting food intake by creating a smaller stomach (in the case of gastric bypass), currently it is thought that adaptations in gut‐brain neuroendocrine signaling play a more prominent role in meditating this effect. Gut hormones, the gut microbiota, and bile acids in particular are postulated to partially account for the metabolic benefits of bariatric surgery (Sinclair *et al*, 2018).

Mimicking the effects of bariatric surgery with pharmacological agents is currently one of the most exciting niches in the anti‐obesity medication market. This approach has already succeeded with semaglutide, a stable GLP‐1 analog approved in June 2021 for weight loss management in overweight or obese subjects. When administered as one‐weekly subcutaneous injection at a dose of 2.4 mg over a 1 year of treatment of non‐diabetic, overweight, or obese patients, semaglutide decreased body mass by −14.9% vs. −2.4% in the placebo‐receiving group (Wilding *et al*, 2021). Compared with its predecessor, liraglutide, a GLP‐1R agonist available since 2014, semaglutide‐mediated weight loss is doubled when applying daily doses equivalent to ~ 10% of high‐dose liraglutide (O'Neil *et al*, 2018). Current improvements focus on designing GLP‐1R agonists administered orally and of effectiveness comparable with parenteral formulations (e.g., GLPR‐NPA is in phase II clinical trials at Eli Lilly). Poly‐agonists, unimolecular peptides that target simultaneously GLP1‐R, GIPR, and/or glucagon receptors, are currently studied in the clinic. In a forty‐week phase III trial tirzepatide, a co‐agonist of GLP‐1R/GIPR, at three tested doses presented superior efficacy to lower the levels of glycated hemoglobin relative to semaglutide at 1 mg in a cohort of patients with type 2 diabetes and excess weight (Frías *et al*, 2021). In the same trial, tirzepatide decreased body weight ≥15% in 15–40% of patients versus 9% in semaglutide‐treated group (Frías *et al*, 2021). Interestingly, combined gut hormone treatment in the form of GLP‐1, oxyntomodulin and PYY (GOP) was shown to cause comparable reductions in body weight and glycemic control to bariatric surgery over the course of 4 weeks in obese and diabetic/pre‐diabetic patients, but to have markedly different effects on the plasma and urinary metabolome (Jones *et al*, 2022). Similarly, combined liraglutide and PYY treatment for 4 weeks in diet‐induced obese rats had little impact on the hypothalamic transcriptome unlike bariatric surgery despite causing similar weight loss (Dischinger *et al*, 2022). These intriguing findings suggest that gut hormones fail to fully recapitulate all the metabolic effects of bariatric surgery and require further study.

A concept of GLP‐1R and glucagon receptor (GlcgR) dual agonists assumes employment of additional mechanism to reduce body weight (i.e., increase in energy expenditure) with minimalization the risk of hyperglycemia (Kleinert *et al*, 2019). A phase IIb trial with cotadutide, a GLP‐1R/GlcgR co‐agonist, was accomplished with a decrease in body weight and hepatic lipid content compared with placebo (Nahra *et al*, 2021). GLP‐1R/GIPR/GlcgR triagonist LY3437943 (GGG) is in an early stage of clinical trials. Thus, incretin‐based therapy offers a previously unachievable efficacy of weight loss with a pharmacological approach.

## Conclusions and future directions

The limited long‐term success in weight loss by inhibiting dietary lipid absorption, which is in common with several other pharmacotherapies aiming to achieve substantial sustained weight loss, requires a critical appraisal. When tipping the energy balance to promote weight loss, we need to take into account that energy intake, energy storage, and energy expenditure are tightly entangled. Physiological regulation rules energy balance according to the settling point for body weight and body composition. For this reason, obese individuals, even when compliant with the standard recommendation to eat less and increase physical activity, recruit counteracting physiological mechanisms to defend their set point of body mass. Further, in response to caloric restriction, total energy expenditure is decreased more than predicted based on body composition (Rosenbaum & Leibel, 2010; Lam & Ravussin, 2016; Hall, 2018), and concomitantly, it does not increase in direct proportion to the intensity of exercise (Westerterp, 1998). It should also be considered that a negative energy balance by pharmacotherapy, as exemplified by SGLT2 inhibitor‐mediated chronic glycosuria, might lead to compensatory increase in energy intake (Ferrannini *et al*, 2015). On the other hand, reduction in dietary fat absorption by orlistat did not alter resting energy expenditure in patients receiving standardized diet (Karhunen *et al*, 2000). Accumulating evidence suggests that individuals differ in their susceptibility to develop such adaptive responses to negative or positive energy balance, characterized by a more spendthrift or thrifty metabotypes (Piaggi, 2019). In this respect, the underlying molecular mechanisms of substantial inter‐individual variation in fecal fat excretion should be scrutinized and considered for the development of a personalized pharmacotherapy. Moreover, the complexity and redundancy of energy flux regulation means that both sides of the energy balance equation need to be targeted. The latter is most promising, as demonstrated by the unprecedented efficacy of the GLP‐1 receptor agonist in inducing weight loss (Smits & Van Raalte, 2021; Wilding *et al*, 2021). Nevertheless, the development of combined therapeutics targeting lipid processing machinery in the intestine and molecular pathways regulating other aspects of metabolic homeostasis might provide better options for the treatment of obesity and associated diseases. Finally, several studies suggest that components of the machinery responsible for lipid absorption in the intestine are implicated in the development of IBD or colorectal cancer. Similarly, the impact of the gut flora on lipid absorption has only recently started to be appreciated. In the future, a detailed investigation of these aspects might open new avenues for the development of novel therapies for multiple metabolic and inflammatory disorders (Fig 1).

## Disclosure and competing interests statement

The authors declare that they have no conflict of interest.

**Pending issues**

1. Which factor(s) coordinate the destination of dietary fats in enterocyte for the secretory pathway (CM synthesis), intracellular storage (LD synthesis), or energy production (FA oxidation) in different lipid supply conditions (fasting vs. postprandial period)? Are the stimuli of neuronal or endocrine origin? Is the central nervous system a superior regulator?
2. What are the consequences of lipid overload for intestinal tissue homeostasis? Can exhaustion of mechanisms aimed at FAs disposal/neutralization (CM, LD synthesis, mitochondria oxidation) in enterocyte—and resulting in elevated FAs concentration—be responsible for lipotoxic cell damage? Does lipotoxicity underlie inflammation‐associated diseases, for example, colorectal cancer and inflammatory bowel disease?
3. How exactly does gut microflora interfere with the lipid processing machinery in the intestine? How the host–microbe interactome can be targeted for anti‐obesity therapeutic use?
